# The fission yeast cell wall stress sensor-like proteins Mtl2 and Wsc1 act by turning on the GTPase Rho1p but act independently of the cell wall integrity pathway

**DOI:** 10.1002/mbo3.113

**Published:** 2013-07-30

**Authors:** Sandra Cruz, Sofía Muñoz, Elvira Manjón, Patricia García, Yolanda Sanchez

**Affiliations:** Instituto de Biología Funcional y Genómica, CSIC/Universidad de Salamanca and Departamento de Microbiología y Genética, Universidad de SalamancaC/Zacarías González s/n., Salamanca, Spain

**Keywords:** Cell wall, cell wall sensor, fission yeast, MAPK pathway, RhoGTPases, stress

## Abstract

Sensing stressful conditions that affect the cell wall reorganization is important for yeast survival. Here, we studied two proteins SpWsc1p and SpMtl2p with structural features indicative of plasma membrane-associated cell wall sensors. We found that Mtl2p and Wsc1p act by turning on the Rho1p GTPase. Each gene could be deleted individually without affecting viability, but the deletion of both was lethal and this phenotype was rescued by overexpression of the genes encoding either Rho1p or its GDP/GTP exchange factors (GEFs). In addition, *wsc1*Δ and *mtl2*Δ cells showed a low level of Rho1p-GTP under cell wall stress. Mtl2p-GFP (green fluorescent protein) localized to the cell periphery and was necessary for survival under different types of cell wall stress. Wsc1p-GFP was concentrated in patches at the cell tips, it interacted with the Rho-GEF Rgf2p, and its overexpression activated cell wall biosynthesis. Our results are consistent with the notion that cell wall assembly is regulated by two different networks involving Rho1p. One includes signaling from Mtl2p through Rho1p to Pck1p, while the second one implicates signaling from Wsc1p and Rgf2p through Rho1p to activate glucan synthase (GS). Finally, signaling through the mitogen-activated protein kinase (MAPK) Pmk1p remained active in *mtl2*Δ and *wsc1*Δ disruptants exposed to cell wall stress, suggesting that the cell wall stress-sensing spectrum of *Schizosaccharomyces pombe* sensor-like proteins differs from that of *Saccharomyces cerevisiae*.

## Introduction

The fungal cell wall is a key defense to withstand environmental adversities and is a potential target for antifungal agents (Heinisch 2005). The *Schizosaccharomyces pombe* cell wall mainly consists of an outer layer rich in galactomannoproteins (14%) and an inner layer of β-1,3-glucans (55%) and α-1,3-glucans (28%), all of which form a large complex (Osumi et al. 1998; Pérez and Ribas 2004). The cell wall is constantly remodeled; it must be loosened to allow expansion during periods of polarized growth, while it needs to be constrained when cells are growing under poor substrate conditions. Its composition also changes upon stress such as heat shock, osmotic changes, destabilizing agents (glucanases or antibiotic agents), or mutations in cell wall genes (Latgé 2007).

Although the structural components of the cell wall have been extensively studied in *S. pombe*, the molecular mechanism by which stresses are detected on the surface and transmitted into the cell remains unknown. In fission yeast, synthesis of the two main polymers β-(1,3)- and α-(1,3)-glucans is regulated through the coordinated action of Rho1p and Rho2p, both of them GTPases of the Rho family (García et al. 2006b; Perez and Rincón 2010). Rho1p participates directly in the production of new cell wall by functioning as the regulatory subunit of β-1,3 glucan synthase (GS), and it also binds and activates the protein kinases of the PKC family, Pck1p and Pck2p, both necessary for the maintenance of cell integrity (Arellano et al. 1999b; Sayers et al. 2000). Rho1p regulation is carried out through its GEF/GAP (GTPase-activating proteins) proteins that turn the GTPase on and off within a specific spatiotemporal context (García et al. 2006b). Rgf1p is a Rho1p GEF that activates the β-GS complex containing the catalytic subunit Bgs4p and is required for the actin reorganization necessary for cells to change from a monopolar to a bipolar growth mode during NETO (New End Take Off) (García et al. 2006a). Rgf1p also signals upstream from the Pmk1 mitogen-activated protein kinase (MAPK) pathway led by Pck2p and Rho2p (Garcia et al. 2009a). Rgf2p (*Rho gef2*) performs an essential function during the sporulation process and a secondary function redundant with Rgf1p during polarized growth (García et al. 2009b). Rgf3p is essential for cell integrity and specifically regulates the synthesis of β-(1,3)-glucan of the division septum through Rho1p (Tajadura et al. 2004; Morrell-Falvey et al. 2005). The GAPs, Rga1p, Rga5p, and Rga8p, are negative regulators of Rho1p but little is known about their contribution to the spatial regulation of Rho1p and β-(1,3)-glucan biosynthesis (Nakano et al. 2001; Calonge et al. 2003; Yang et al. 2003).

Rho2p regulates α-(1,3)-glucan synthesis through Pck2p. Rho2p binds to Pck2p and both are required for the activity and proper localization of the α-(1,3)-GS catalytic subunit (Mok1p/Ags1p) (Arellano et al. 1999b; Calonge et al. 2000). To date, there is no positive modulator that specifies Rho2p function in α-glucan biosynthesis, although a mutant in Rga2p, a GAP for Rho2p, shows increased levels of α-(1,3)-glucan (Villar-Tajadura et al. 2008). Rho2p and Pck2p also regulate gene expression via the Pmk1p MAPK cell integrity signaling pathway (Ma et al. 2006; Barba et al. 2008).

In order to adapt to external conditions, stressed *S. pombe* cells switch on the stress-induced cell wall biosynthetic machinery, and in the long term they modify the relative amount or the cross-linking of their cell wall polymers. For example, osmotic stress and some antifungal agents arrest tip growth and induce the deposition of abnormal cell wall material at the tips, perhaps covering weakened areas of the cell wall (Robertson and Hagan 2008). In other cases, defects in the synthesis of β-glucan caused by mutations in the β-GS genes *bgs1*, *bgs3,* or *bgs4*- induce compensatory mechanisms that reinforce the cell wall by an increase in α-1,3-glucan levels (Martin et al. 2003; Cortes et al. 2005).

In this study, we focused on cell surface proteins able to detect cell wall stress in fission yeast. One candidate for this type of protein is SpWsc1p, first described by Willer et al. (2005). However, to date neither SpWsc1p nor any other plasma membrane receptors have been shown to activate the cell integrity pathway in *S. pombe*. In *Saccharomyces cerevisiae* sensing is achieved by two groups of transmembrane (TM) proteins, the cell wall integrity and stress response component (WSC) family (Slg1/Wsc1p, Wsc2p, and Wsc3p) and the Mid2p-Mtl1p pair (Rodicio and Heinisch 2010; Jendretzki et al. 2011; Levin 2011). These families of sensors appear to respond to different types of stimulation, as suggested by the differential sensitivity to external stress exhibited by mutants in these genes (Verna et al. 1997; Reinoso-Martín et al. 2003; Vilella et al. 2005; Wilk et al. 2010). Their overall structures are similar in that they possess small C-terminal cytoplasmic domains, a single TM domain, and a periplasmic domain rich in Ser/Thr residues. These Ser/Thr-rich (STR) regions are highly O-mannosylated, probably resulting in the extension and stiffening of the polypeptide (Rajavel et al. 1999; Lommel et al. 2004). Accordingly, these proteins have been proposed to function as mechanosensors, their ectodomains acting as rigid probes of the extracellular matrix (Rajavel et al. 1999; Philip and Levin 2001; Dupres et al. 2009). In *S. cerevisiae* these sensors activate Rho1p by recruiting the Rom2p Rho1p GEF and the peripheral plasma membrane protein Zeo1p (Philip and Levin 2001; Green et al. 2003; Vay et al. 2004). Rho1p is the hub of many signaling pathways and suppression analyses using the sensors have indicated that Mid2p and Wsc1p signaling through Rho1p leads to different outputs, with Mid2p-activated Rho1p signaling through Pkc1p and Wsc1p-activated Rho1p stimulating Fks1p and Pkc1p (Schmitz et al. 2002; Sekiya-Kawasaki et al. 2002; Green et al. 2003; Reinoso-Martín et al. 2003; Bermejo et al. 2010).

Here, we report that the simultaneous depletion of SpWsc1p and SpMtl2p is lethal, revealing the complementary functions of these sensors. Mild overexpression of *rho1*^+^ fully rescues a double *mtl2*Δ *wsc1*Δ mutant, suggesting that Wsc1p and Mtl2p share an essential function as Rho1p activators during vegetative growth.

## Experimental Procedures

### Media, reagents, and genetics

The genotypes of the *S. pombe* strains used in this study are listed in Table 1. Standard *S. pombe* media and genetic manipulations were employed (Moreno et al. 1991). Caspofungin acetate (Csp) was stored at −20°C in a stock solution (2.5 mg/mL) in H_2_O and was added to the media at the corresponding final concentration after autoclaving. Crosses were performed by mixing appropriate strains directly on malt extract agar plates. Recombinant strains were obtained by tetrad analysis or the “random spore” method. For overexpression experiments using the *nmt1* promoter, cells were grown in edinburgh minimal medium containing 15 μmol/L thiamine up to logarithmic phase. Then, the cells were harvested, washed three times with water, and inoculated in fresh medium (without thiamine) at an OD_600_ = 0.01.

**Table 1:** *Schizosaccharomyces pombe* strains used in this work

Strains	Genotypes
YSM180	h^−^ 972
YS64	h^−^ *leu1-32 ade6M210 ura4D-18 his3D1*
HVP54^a^	h^−^ *leu1-32 ade6M210 ura4D-18*
VT14	h^−^ *rgf1::his3*^*+*^*, leu1-32, ade6M210, ura4d18, his3D1*
YS165	h^+^/h^−^ *leu1-32*/*leu1-32 ade6M210*/*ade6M216 ura4D-18*/*ura4D-18 his3D1/his3D1*
GRG8	h^−^ *wsc1::ura*^*+*^ *leu1-32 ade6M210 ura4D-18 his3DI*
SC136	h^−^ *wsc1::his3*^*+*^ *leu1-32 ade6M210 ura4D-18 his3DI*
GRG15	h^−^ *wsc1::kan*^*+*^ *leu1-32 ade6M210 ura4D-18 his3DI*
YS1220	h^−^ *mtl2::his3 ura4d18 leu1-32 ade6M210 his3DI*
SC33	h^−^ *kan-81xnmt1-mtl2*^*+*^ *leu1-32 ura4D18 his3D ade6M210*
SC92	h^−^ *mtl2::kan ura4d18 leu1-32 ade6M210 his3DI*
PPG160^b^	h^−^ *rho1:HA leu1-32*
MS192^c^	h^−^ *spm1::LEU2 leu1-32 ura4D18*
SC123	h^+^ *mtl2::his3*^+^ *rho1:HA leu1-32 his3DI ade6M*210
SC142	h^+^ *wsc1::his3*^+^ *rho1:HA leu1-32 his3DI ade6M*210
SC80	h^+^ *kan-P81xnmt1-mtl2*^*+*^ *wsc1::ura*^*+*^ *leu1-32 ura4D18 his3D ade6M210*
MI200^d^	h^+^ *pmk1-HA6his: ura4*^*+*^ *ade6M216 leu1-32 ura4d18*
SC19	h^−^ *mtl2::his3 pmk1-HA6his:ura*^*+*^ *ura4D18 leu1-32 ade6M210*
SC141	h^−^ *wsc1::his3 pmk1-HA6his:ura*^*+*^ *ura4D18 leu1-32 ade6M210*
PG65	h^−^ *rgf1::kan, leu1-32 ade6M210 ura4d18, his3D1*
VT88	h^−^ *P81nmt-rgf3*^+^*:ura4*^+^ *leu1-32 ade6M210 ura4D-18 his3DI*
SC167	h^−^ *mtl2-GFP:ura4*^*+*^ *leu1-32 ade6M210 ura4D-18 his3DI*
GRG45	h^−^ *wsc1-GFP:ura4*^*+*^ *leu1-32 ade6M210 ura4D-18 his3DI*
SC255	h^−^ *wsc1-GFP:ura4*^*+*^ *mtl2::kan his3D1 leu1-32 ade6M210*
SC252	h^−^ *mtl2-GFP:ura4*^*+*^ *wsc1::kan his3D1 leu1-32 ade6M210*
JCR962^e^	h^*+*^ *crn1GFP:Kan ade-6M leu1-32 ura4D18*
AR606^f^	h^−^ *aur-mCherry-atb2*^*+*^ *leu1-32 ura4D18*
SC240	h^*−*^ *wsc1-GFP:ura4*^*+*^ *aur-mCherry-atb2*^*+*^ *ura4D18*
SC242	h^−^ *mtl2-GFP:ura4*^*+*^ *aur-mCherry-atb2*^*+*^ *ura4D18*
SC173	h^+^ *kan-P81xnmt1-rgf1*^*+*^ *wsc1:: kan*^*+*^ *leu1-32 ura4D18 his3DI*
GRG33	h^−^ *kan-P81Xnmt-rgf1*^*+*^ *rgf2::ura4*^*+*^ *leu1-32 ura4D-18 his3D1 ade6M210*
PG1	h^−^ *rgf2::ura4*^+^ *his3d1 ura4D18 leu1-32 ade6M210*
SC98	h^−^ *wsc1::kan*^*+*^ *rgf2::ura4*^+^ *his3D1 ura4D18 leu1-32 ade6M210*
PG40	h^−^ *rgf1::his3*^*+*^ *leu1-32::rgf1*^*+*^*-GFP:leu1*^*+*^ *his3D1, ura4D18*
SC165	h^−^ *rgf1::his3*^*+*^ *leu1-32::rgf1*^*+*^*-GFP:leu1*^*+*^ *wsc1::kan*^*+*^ *his3D ura4D18*
SC136^g^	h^−^ *wsc1::his3*^*+*^ *leu1-32 ade6M210 ura4D-18 his3DI +* (*p41xnmt1-rgf2-GFP*)
SC238	h^−^ *rgf3::ura4*^+^ *leu1*^*+*^*::EGFP- rgf3*^+^ *leu1-32 ade6M210 ura4D-18*, *his3DI*
SC198	h^−^ *wsc1::kan*^+^ *rgf3::ura4*^+^ *leu1*^*+*^*::EGFP- rgf3*^*+*^ *leu1-32 ade6M210 ura4D-18 his3DI*

All strains were generated in this study except for strains with label^a^ from H. Valdivieso (IBFG, University of Salamanca), label^b^ from Pilar Perez (IBFG, University of Salamanca), label^c^ from J. Cooper (Washington University, St. Louis), label^d^ from J. Cansado (University of Murcia), label^e^ from F. Chang (Columbia University, NY), and label^f^ from T. Toda (London Research Institute). Strain labeled^g^ was transformed with plasmid p41xnmt1-Rgf2-GFP.

### Disruption of the *wsc1*^+^ and *mtl2*^+^ genes

The *wsc1::ura4*^+^ disruption construct was obtained in a two-step process. The 5′ noncoding region of the *wsc1*^+^ open reading frame (ORF) (nucleotides [nt] −1010 to +34) was amplified by polymerase chain reaction (PCR), inserting the *Xho*I and *Hind*III sites (one at each end), and was ligated into the same sites of the SK-*ura4*^+^ vector. The 3′ flanking region of the *wsc1*^+^ ORF (nt +1027–2100) was amplified by PCR, inserting the *Bam*HI and *Not*I sites as above, and was cloned into the same sites of pSK-*ura*^+^ with the 5′ end, to yield pCL1. *wsc1*^+^ gene disruption was accomplished using the 3.8-kb fragment from pCL1 cut with *Xho*I and *Not*I and transforming the YS64 haploid strain. Transformants were replica plated five times consecutively on YES medium to eliminate cells that had not integrated the construct. Integration was analyzed by PCR using the following oligonucleotides: M13 (5′-TTACCTAAGGCCACCAG-3′) in the *ura4*^+^ gene and 5′-comp-wsc1 (5′-CGTGGGTACTTCGACATG-3′) upstream from nucleotide +1153, and hence external to the disruption cassette. To make the *wsc1::kan* disruption construct (pRZ3), the uracil marker from plasmid pCL1 was excised and replaced by the kanamycin marker. *wsc1*::*kan* disruptants (GRG14 and GRG15) were obtained as above, tested for stability, and analyzed by PCR. To obtain the *mtl2::his3*^+^ disruption construct, the 5′ noncoding region of the *mtl2*^+^ ORF (nucleotides [nt] −1425 to −6) was obtained by PCR, inserting the *Apa*I and *Sal*I sites (one at each end), and was ligated into the same sites of the pSK-his3 vector to yield pYS13. The 3′ flanking region of the *wsc1*^+^ ORF (nt +4433 to +5697) was obtained by PCR, inserting the *Pst*I and *Not*I sites as above, and was cloned into the same sites of pYS13 to yield pYS2. *mtl2*^+^ gene disruption was accomplished using the 5.4-kb fragment from pYS2 cut with *Apa*I and *Not*I and transforming the YS64 strain. Correct integration was analyzed by PCR using the following oligonucleotides: M22 (5′-GTGTTCGCTAATTGCGA-3′) in the *his3*^+^ gene and Mid2-ext-5′ (5′-GTTGCTCTCATCCGTTG-3′) upstream from nucleotide −1290 and therefore external to the disruption cassette. For the *mtl2::kan* disruption construct (pSC13), the 5′ and the 3′ noncoding regions of *mtl2*^+^ were subcloned into the vector pKS-kan, proceeding as above.

### Plasmid and DNA manipulations

To make pCL10 (pAL*wsc1* ORF), a *Nco*I-*Bam*HI fragment containing the *wsc1*^+^ ORF was obtained by PCR and was subcloned into the same sites of pCL1 (described above). Based on the *wsc1*^+^ sequence, we designed oligonucleotides 400 bp apart and sequenced the entire ORF of four different clones. To tag Wsc1p at the C-terminus with GFP (engineered with eight alanines at the N-terminus) and with the triple repeat of the influenza virus hemagglutinin epitope (HA) (Craven et al. 1998), pAL-*wsc1*^+^ (pCL10) was modified by site-directed mutagenesis. Site-directed mutagenesis was also used to create a *Not*I site immediately before the TAA stop codon of *wsc1*^+^ (pCL14). The GFP and HA epitopes were inserted in-frame at the *Not*I site of pCL14 to create pRZ11 and pRZ12, respectively. Strains with a genomic copy of *wsc1*^+^ tagged with GFP were obtained by one-step gene replacement. pRZ11 was modified to create a *Sma*I behind the *wsc1*^+^ ORF, allowing the insertion of the *ura4*^+^ marker at that site (pRZ21). A *Spe*I-*Bgl*II fragment of pRZ21 was used to transform a wild-type strain YS64, and correct integration of the fusion protein was analyzed by PCR. For *wsc1*^+^ overexpression, pCL10 (pAL-*wsc1*^+^) was modified by site-directed mutagenesis, introducing *Xho*I and *Sma*I sites flanking the *wsc1*^+^ ORF and thus creating pRZ15. The *wsc1*^+^ ORF from pRZ15 was cloned into the same sites of pREP3X and pREP81X, thus affording pRZ16 and pRZ17, respectively. To overexpress HA-tagged *wsc1*, a *Nco1*I-*Sma*I fragment containing the *wsc1*^+^ gene tagged with the HA epitope from plasmid pRZ12 (described above) was ligated into the *Nco*I-*Eco*RV sites of plasmid pJCR-L3x (Moreno et al. 2000). pSC2 (pAL *mtl2* ORF) was obtained by gap repair. Upstream and downstream flanking sequences from *mtl2*^+^ (from pYS2) were subcloned in pALKS. The plasmid was linearized with the 5′ and 3′ fragments at the ends and used to transform the wild-type haploid strain (YS64). The gap in the plasmid was repaired using the chromosomal sequences and the plasmids were recovered from the yeasts. Transformants were replica plated five times consecutively on YES medium and those able to lose the plasmid were selected. To tag Mtl2p at its C-terminus with the GFP, pSC2 was modified by site-directed mutagenesis. We created a *Not*I site immediately before the termination codon (pSC5). The GFP epitope was inserted in-frame at the *Not*I site of pSC5. pAL-*mtl2*^*+*^*GFP* (pSC7) fully complemented the *mtl2*Δ phenotypes. Strains with a genomic copy of *mtl2*^+^ tagged with GFP were obtained by one-step gene replacement. pSC9 was modified to create a *Sma*I at the 3′end of the *mtl2*^+^ ORF, allowing the insertion of a *ura4*^+^ marker at that site (pSC19). A 5.7-Kb *Spe*I-*Bst*XI fragment from pSC19 was used to transform a YS64 wild-type strain.

### Cell wall analyses

Enzyme preparations and GS assays were performed essentially as described previously (Martin et al. 2003). Cell extracts were obtained from early log-phase cells grown in YES or minimal medium (MM) as indicated for each case. For the labeling and fractionation of cell polysaccharides in a standard protocol, exponentially growing cultures of *S. pombe* cells were supplemented with U-[^14^C]-glucose (3 μCi/mL) and incubated for an additional 6 h at 28°C. To label cells overproducing *wsc1*^+^ *and mtl2*^+^, cultures were induced for 14 h in the absence of thiamine before addition of U-[^14^C]-glucose (3 μCi/mL) and incubated for an additional 10 h. In both cases the radioactivity incorporated in the cell wall polysaccharides was measured as described (Tajadura et al. 2004).

### Pull-down assays for GTP-bound Rho proteins

The pGEX-C21RBD (rhotekin-binding domain) expression vector (Reid et al. 1996) was used to transform *Escherichia coli* cells. The fusion protein was produced according to the manufacturer′s instructions and immobilized on glutathione-Sepharose 4B beads (GE Healthcare, Uppsala, Sweden). After incubation, the beads were washed several times and the bound proteins were analyzed by SDS-PAGE (sodiumdodecyl sulfate polyacrylamide gel electrophoresis) and Coomassie staining. The amount of GTP-bound Rho proteins was analyzed using 50 mL cultures of wild-type, *wsc1*Δ, and *mtl2*Δ cells containing HA-*rho1*^+^ expressed from its own promoter and performed as in Garcia et al. (2009a).

### Purification and detection of activated Pmk1p-HA6H after different types of stress

Experiments designed to investigate Pmk1 activation under stress were performed using log-phase cell cultures (OD_600_ of 0.5) growing at 28°C in YES medium and the appropriate stress treatment. Cell homogenates were prepared under native conditions employing chilled acid-washed glass beads and lysis buffer (10% glycerol, 50 mmol/L Tris-HCl, pH7.5, 150 mmol/L NaCl, 0.1% Nonidet P-40, plus a specific protease inhibitor cocktail, 100 μmol/L p-aminophenyl methanesulfonyl fluoride, leupeptin, and aprotinin). The lysates were cleared by centrifugation at 13,000*g* for 10 min and Pmk-HA6H was purified with Ni^2+^-NTA-agarose beads (Novagen, EMD Chemicals, San Diego, CA). The purified proteins were loaded on 10% SDS-PAGE gels, transferred to an Immobilon-P membrane (Millipore, Bedford, MA), and blotted to detect Pmk1-HA with 1:5000 diluted 12CA5 mAb as primary antibody (Roche Diagnostics, Mannheim, Germany) and with polyclonal rabbit anti-phospho-p42/44 antibodies (1:2500) (Cell Signaling, Merck/Millipore, Germany). The immunoreactive bands were revealed with anti-mouse or anti-rabbit HRP secondary antibodies (Bio-Rad Laboratories, Hercules, CA) and the enhanced chemiluminescence detection kit (Amersham Biosciences, Piskataway, NJ).

### Microscopy and image analysis

Cell samples were visualized using an Olympus IX71 microscope equipped with a personal Delta Vision system and a Photometrics CoolSnap HQ2 (Photometrics, Tucson, AZ) monochrome camera. Stacks of seven *z*-series sections were acquired at 0.2-μm intervals. All fluorescence images are maximum two-dimensional (2D) projections of *z*-series and were analyzed using deconvolution software from Applied Precision (GE Healthcare Company, Issaquah, WA). Measurements were made from micrographs using the IMAGEJ (National Institutes of Health, Bethesda, MD) or METAMORPH (Molecular Devices, Sunnyvale, CA) programs. For calcofluor white (Cfw) fluorescence, cells were harvested (1 mL), washed once, and resuspended in water with 20 μg/mL of Cfw at room temperature.

## Results

**Figure 1:**
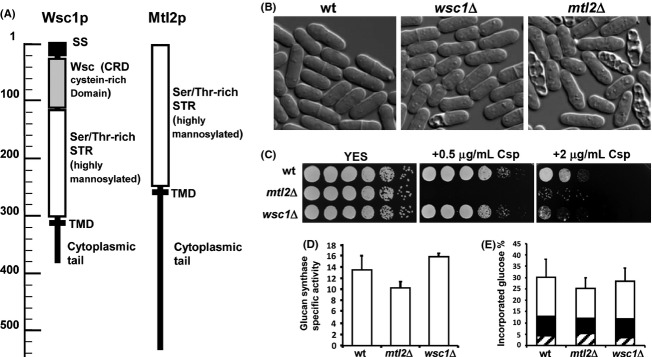
Morphology and growth phenotypes of wild-type (HVP54) and mutant strains *wsc1*Δ (GRG15) and *mtl2*Δ (SC92). (A) Schematic models of the Wsc1p and Mtl2p cell wall integrity sensors (http://smart.embl-heidelberg.de/). SS, signal sequence; CRD, cysteine-rich domain; STR, serine/threonine-rich region; TMD, plasma membrane-spanning domain. (B) Differential interference-contrast (DIC) micrographs of log-phase cells of the indicated strains grown in YES liquid medium at 28°C. (C) Equal numbers of cells of each strain were diluted and (4 × 10^4^, 2 × 10^4^, 2 × 10^2^, and 2 × 10^1^ cells, respectively) were spotted on YES plates in the absence or in the presence of 0.5 and 2 μg/mL of the antifungal agent Csp (CANCIDAS™). Colony formation was analyzed after 2–3 days of incubation at 28°C. (D) The same cells as in (B and C) were grown on YES liquid medium at 28°C and the GS activity of cell extracts was analyzed at 30°C. Values are the specific activity average, calculated from three independent extracts and error bars represent standard deviations (SDs). (E) Cell wall composition in *wsc1*Δ and *mtl2*Δ cells. Cells were grown in YES medium and [^14^C]-glucose was added 6 h before the cells were harvested. The relative levels of [^14^C]-glucose radioactivity incorporated into each cell wall polysaccharide (black α–glucan, white β-glucan, and shaded galactomannan) are shown for the strains indicated above. Values are the means arising from at least three independent experiments (duplicate samples). SDs for total carbohydrate values are shown.

To identify upstream components of the Rho1p/Pck1p/Pck2p pathway that regulate cell integrity in *S. pombe* we performed a blast search (Altschul et al. 1997) of the *S. pombe* genome database at the Sanger Institute with protein sequences of the *S. cerevisiae* ScWsc1p and ScMid2p. We selected three ORFs, SpWsc1p (SPBC30B4.01c) and two sequence orphans with certain similarity to ScMid2p, SpMtl1p (SPBC215.13) and SpMtl2p (SPAC11G7.01). However, we could not designate any of these proteins as SpMid2p because fission yeast Mid2p was already described as a component of the septin ring (Berlin et al. 2003). For this study, we considered SpWsc1p and SpMtl2p (Mid two like 2), both containing a single TM domain and a cytoplasmic tail, while SpMtl1p (Mid two like 1) will be described elsewhere. SpWsc1p has an overall protein sequence similarity of 48% and an identity of 30% with ScWsc1p, and SpMtl2p has 55% similarity and 33% identity with ScMid2p. SpWsc1p and SpMtl2p are single-pass TM proteins of 374 and 536 amino acids (aa), respectively, and their extracellular regions have a highly domain (Fig. 1A). This region is the target of O-mannosylation reactions, as reported for SpWsc1p, and has been proposed to provide them with a rod-like structure (Willer et al. 2005). The TM is followed by a small intracellular C-terminus in SpWsc1p (aa 318–374), and a very long one in SpMtl2p (aa 268–536) (Fig. 1A). There is no significant sequence similarity between the cytoplasmic tails. Wsc1p also shows a signal peptide (aa 1–20) and a putative Wsc domain near the external amino-terminal end (aa 34–109). Wsc domains are cysteine-rich domains (CRD) (Fig. 1A), presumed to anchor these proteins to the cell wall but also to mediate their clustering upon stress (Heinisch et al. 2010).

### Wsc1p and Mtl2p participate in the regulation of cell wall integrity in *S. pombe* cells

To investigate the function of *wsc1*^+^ and *mtl2*^+^, we created strains defective in either *wsc1* or *mtl2* by replacing the *wsc1* ORF with the *ura4*^+^ marker and the *mtl2* ORF with the *his3*^+^ marker (see Experimental Procedures). The *wsc1*Δ and *mtl2*Δ mutants grew well under standard growth conditions at both 28 and 37°C and entered the stationary phase at the same time as the wild-type cultures. *wsc1*Δ and *mtl2*Δ cells did not exhibit any evident morphological changes as judged by light microscopy, but ∼8% of the cells in the *wsc1*Δ mutant and 15% of the cells in *mtl2*Δ were lysed (Fig. 1B). This phenotype was similar to that observed in *rgf1*Δ mutants and in cells depleted for Rho1p (Arellano et al. 1997; García et al. 2006a), and it was suppressed by the addition of 1.2 mol/L sorbitol (data not shown). We then wondered whether the mutants had a defect in cell wall integrity. We examined the sensitivity of *mtl2*Δ and *wsc1*Δ null mutants to different concentrations of Csp (CANCIDAS™, Merck Ltd), a lipopeptide antibiotic that inhibits β-(1,3)-glucan biosynthesis (Garcia-Effron et al. 2009). As shown in Figure 1C, *mtl2*Δ cells were unable to grow on plates supplemented with 0.5 μg/mL Csp, whereas the wild-type cells were able to withstand concentrations of up to 5 μg/mL. *wsc1*Δ cell growth was inhibited above 2 μg/mL of Csp (Fig. 1C). We found that GS activity was slightly reduced in *mtl2*Δ null cells (Fig. 1D). Moreover, when we looked at the cell wall composition of *mtl2*Δ mutants we found a decrease in the total amount of glucose incorporated in the cell wall as compared with wild-type cells (30% in wild-type cells and 25% in *mtl2*Δ). The difference was mainly due to a decrease in the β–glucan content (17% in the wild type and 13% in the *mtl2*Δ) (Fig. 1E). The GS activity and the cell wall composition were very similar in *wsc1*Δ and wild-type cells (Fig. 1D and E). These results suggest that the *mtl2* null mutant cells lose their integrity, probably due to defects in cell wall remodeling in response to damage. We also examined the involvement of Mtl2p and Wsc1p across a range of stress responses. Deletion of *mtl2*^+^ rendered cells hypersensitive to caffeine, vanadate, NaCl, H_2_O_2_, and SDS, while the growth of *wsc1*Δ cells was much less affected by the stresses (see Fig. S1).

### Wsc1p and Mtl2p are functionally redundant

We next looked at the effect of the mutations on mating and sporulation. The mating rate was not affected in *mtl2*Δh^+^ × *mtl2*Δh^−^ or *wsc1*Δh^+^ × *wsc1*Δh^−^ homozygous crosses, and the *mtl2*Δ or *wsc1*Δ segregants were obtained readily. To test the phenotype resulting from the complete deletion of the *wsc1* and the *mtl2* genes, we analyzed tetrads of a *wsc1*::*ura4*^+^ h^−^ × *mtl2*::*his3*^+^ h^+^ cross. We failed to find colonies of the double mutant (*wsc1*Δ*mtl2*Δ). However, microscopic observation of the germinated *wsc1*::*his3*^+^ *mtl2*::*ura*^+^ null spores revealed that they were capable of polarity establishment and appeared to undergo a few cell divisions before growth stopped.

**Figure 2:**
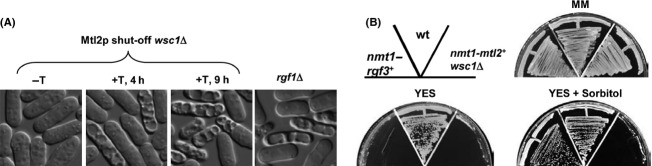
Double disruption of Mtl2p and Wsc1p is essential for cell viability. (A) Depletion of Mtl2p in a *wsc1*Δ background leads to a lysis phenotype similar to the depletion of Rho1p. Lethal phenotype of the *P81 nmt-mtl2wsc1*Δ (SC80) and *rgf1*Δ (PG65) shut-off mutants. Cells grown at 28°C in MM were supplemented with thiamine to repress the *nmt* promoter. For Mtl2p shut-off, Nomarski micrographs were taken before and after the addition of thiamine (4 h and 9 h). To see the *rgf1*Δ “lysis phenotype”, the micrographs were taken from log-phase cells grown in YES. (B) Strains *P81 nmt-rgf3*^+^ (VT88), wild-type (HVP54), and *P81 nmt-mtl2wsc1*Δ (SC80) were streaked onto MM (w/o thiamine), YES and YES plus 1.2 mol/L Sorbitol plates and incubated at 28º for 4 days. The *nmt* promoter is -off- in rich medium (YES) and -on- in MM.

To further characterize the synthetic lethal phenotype of Mtl2p and Wsc1p during vegetative growth, we created a strain *P81nmt*-*mtl2 wsc1*Δ (SC80), deleted for the *wsc1*^+^ gene and with the endogenous *mtl2*^+^ expressed under the control of the *P81nmt* promoter (*P81nmt* is a thiamine-regulatable and reduced-expression-rate promoter). Viable *P81nmt*-*mtl2 wsc1*Δ cells were obtained only when the *81nmt* promoter was induced (Fig. 2B) and osmotic stabilization did not restore viability to the cells under conditions of *mtl2* promoter shut-off (Fig. 2B). Repression of *mtl2*^+^ promoted cell lysis and the cells shrunk without the release of cytoplasmic material. The lysed cell phenotype was analogous to that observed earlier in the *rgf1*Δ mutant (Fig. 2A) and in cells depleted for Rho1p (Arellano et al. 1997) and occurred mainly at the poles, suggesting that the function of Mtl2p and Wsc1p at the tips boundary is more critical than that at the cell wall septum (Fig. 2A). These results indicate that the *mtl2 and wsc1* depletion-mutant phenotypes are very similar to those of Rho1p-depleted cells, suggesting that such mutants are defective in activation of either Rho1p or its downstream effectors**.**

### Wsc1p and Mtl2p are recruited to different areas of the cell surface

**Figure 3:**
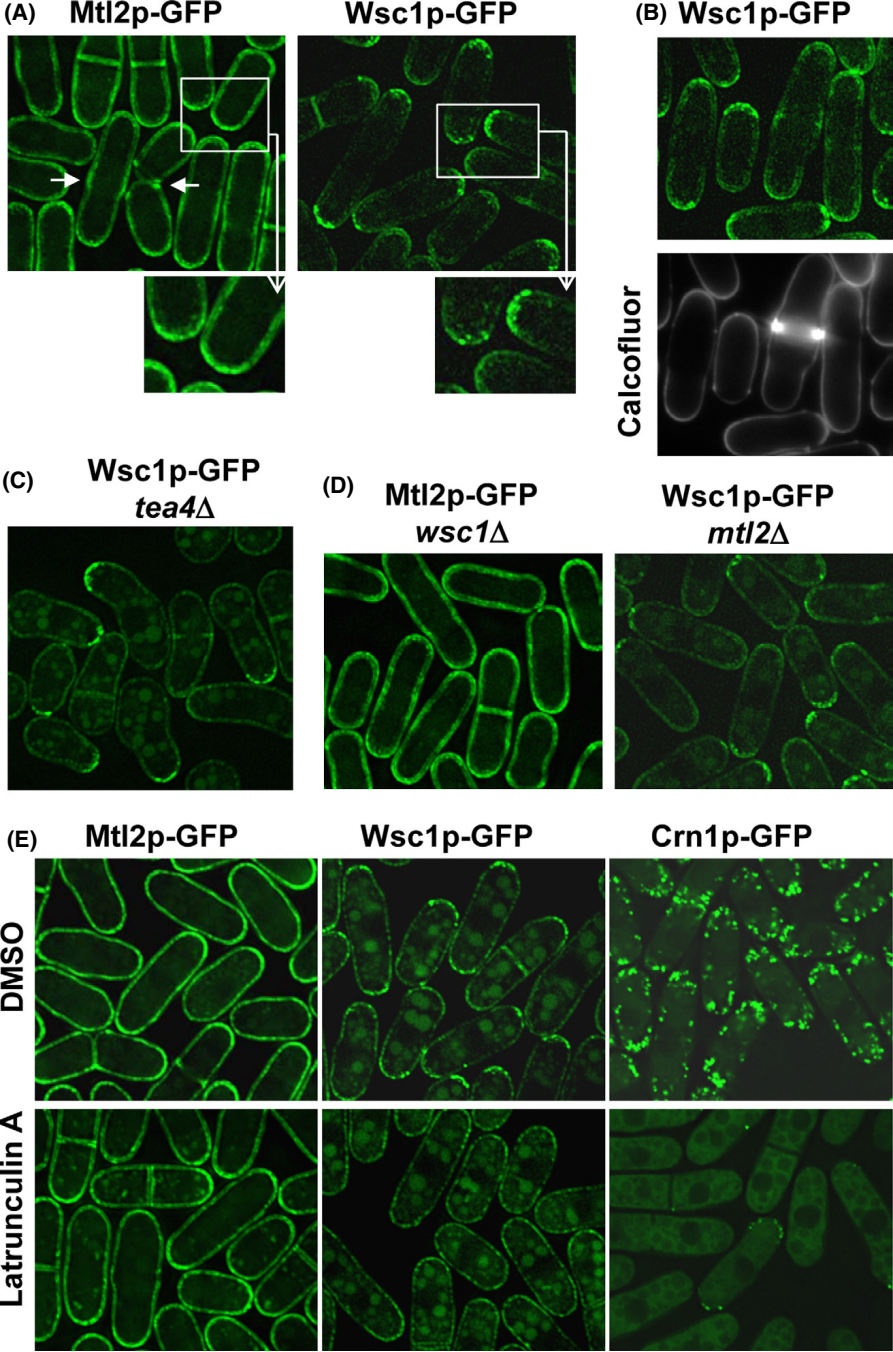
Wsc1p and Mtl2p are recruited to distinct areas of the cell surface. (A) Maximum projections of Mtl2-GFP (SC167) and Wsc1p-GFP (GRG45) localization. The tagged proteins were integrated as a single copy at the endogenous *mtl2 and wsc1* loci, respectively. (B) Wsc1p-GFP (GRG45) and Cfw staining in wild-type cells. Cfw was added at 20 μg/mL, followed by immediate examination of the cells (lower panel). (C) Wsc1p-GFP localizes exclusively to the growing cell end in *tea4*Δ cells. (D) The distribution of Mtl2p-GFP or Wsc1p-GFP was not affected in strains from which the other putative sensor had been deleted. Imaging of Mtl2p-GFP *wsc1*Δ cells shows no increase in the intensity of the Mtl2p-GFP signal upon deletion of *wsc1*^+^ (SC252) (left). Imaging of Wsc1p-GFP *mtl2*Δ cells shows no changes in the localization of Wsc1p-GFP signal upon deletion of *mtl2*^+^ (SC255) (right). (E) Accumulation of Wsc1p-GFP to the poles is dependent on the actin cytoskeleton. Cells expressing Mtl2p-GFP (SC167), Wsc1p-GFP (GRG45), and Crn1p-GFP (JCR962) were treated with dimethyl sulfoxide (upper panels) or 100 μmol/L LatA (lower panels) for 30 min and then analyzed for Mtl2p-GFP, Wsc1p-GFP, and Crn1p-GFP localization, respectively. Maximum projections are shown.

In order to identify the location of Mtl2p and Wsc1p in the cell, the sequences encoding GFP were fused in-frame to their respective genes at the 3′-end encoding the C-terminus, and the native loci were replaced by the tagged copies using a “one-step replacement approach.” Cells carrying Mtl2p-GFP or Wsc1p-GFP responded as wild-type cells to different concentrations of Csp, indicating that the fusion proteins were fully functional. Mtl2p-GFP showed an even membrane distribution with little intracellular signals. We failed to observe a polarized location of Mtl2p-GFP to specific regions of the surface. However, it is worth mentioning that the fluorescence was slightly more intense at the poles and as a belt in the middle area of cells in interphase (Fig. 3A, white arrow). In addition, Mtl2p-GFP was seen at the division scars after cell separation (Fig. 3A). Wsc1p-GFP was found along the entire plasma membrane, but appeared much more concentrated in patches at the cell ends. We also noted that Wsc1p-GFP accumulated in intracellular compartments (Fig. 3C and D). Cfw staining showed that Wsc1p-GFP dots appeared in a single tip when cells were growing in the monopolar mode and in both cell tips when the cells had activated NETO and growth was occurring at both tips (Fig. 3B). To determine whether Wsc1p localization was correlated with the growing tips and depended on the polarity markers, we observed the Wsc1p-GFP localization pattern in cells lacking the end marker Tea4p. These cells do not activate the new end and grow in a monopolar manner (Martin et al. 2005; Tatebe et al. 2005). In *tea4*Δ cells, Wsc1p-GFP localized mainly to the growing tip that was stained with Cfw (Fig. 3C). These results suggest that Wsc1p could form part of a complex with other proteins at growth regions. Consistent with this, the localization of Wsc1p-GFP to cell tips was strongly affected by disruption of the actin filaments with latrunculin A (latA) (Fig. 3E), but was unaffected by acute disruption of microtubules with the microtubule-depolymerizing drug methyl 2-benzimidazolecarbamate (MBC) (Fig. S2). The distribution of Mtl2p-GFP across the whole cell surface was not affected by treatment with either latA or MBC (Figs. 3E, S2). Thus, the localization of Wsc1p-GFP seems to be more dynamic than that seen for Mtl2p-GFP, and might depend on endocytic turnover as shown before for members of the Wsc1p family in *S. cerevisiae* (Piao et al. 2007; Straede and Heinisch 2007; Wilk et al. 2010).

Although Mtl2p-GFP and Wsc1p-GFP had strikingly different distributions, cells that lacked either molecule were viable, yet those that lacked both were dead (see Fig. 2), suggesting either that the association of Mtl2p-GFP or Wsc1p-GFP with some structures is not essential, or that the removal of one putative sensor enables the other to substitute and assume the functions that were normally executed by the missing one. However, we did not find any apparent changes in the distribution and signal intensity of Mtl2p-GFP or Wsc1p-GFP in strains from which the other putative sensor had been deleted (Fig. 3D).

### Mtl2p and Wsc1p control cell wall integrity through Rho1p

**Figure 4:**
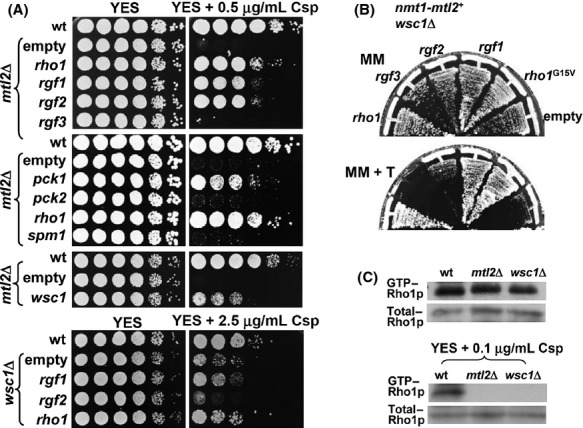
Activation of the Rho1 GTPase switch rescues growth in Mtl2p-Wsc1p-depleted cells. (A) The Csp–hypersensitive growth phenotype of *mtl2*Δ mutants is suppressed by overexpression of *rho1*^+^, *rgf1*^+^, *rgf2*^+^, *pck1*^+^, and *wsc1*^+^. (Top panels) *mtl2*Δ (YS1220) was transformed with pAL (empty vector), pAL-*rho1*^+^, pAL-*rgf1*^+^, pAL-*rgf2*^+^, pAL-*rgf3*^+^, pDB248*pck1*^+^, pDB248*pck2*^+^, pAL-*wsc1*^+^, and pART-*spm1*^+^. Transformants were spotted onto YES and YES plus 0.5 μg/mL of Csp plates as serial dilutions (8 × 10^4^ cells in the left row, and then 4 × 10^4^, 2 × 10^4^, 2 × 10^3^, 2 × 10^2^, and 2 × 10^1^ in each subsequent spot) and incubated at 28°C for 3 days. (Lower panel) *wsc1*Δ (GRG14) was transformed with pAL (empty vector), pAL-*rho1*^+^, pAL-*rgf1*^+^, and pAL-*rgf2*^+^. Transformants were spotted onto YES and YES plus 2.5 μg/mL of Csp plates as serial dilutions as above. (B) The *P81 nmt-mtl2wsc1*Δ (SC80) shut-off mutant was transformed with pAL (empty vector), pAL-*rho1*^+^, pAL-*rgf3*^+^, pAL-*rgf2*^+^, pAL-*rgf1*^+^, and pAL-*rho1*^G15V^ (rho hyperactive allele) and the colonies were streaked onto MM (w/o thiamine) and MM + thiamine plates and incubated at 28°C for 4 days. (C) The level of Mtl2p and Wsc1p modulates the amount of GTP-bound Rho1p in vivo in the presence of Csp. The wild-type (PPG160), *wsc1*Δ (SC142), and *mtl2*Δ (SC123) cells expressing HA-*rho1*^+^ from its own promoter were grown in YES or YES liquid media supplemented with 0.1 μg/mL of Csp for 16 h. Samples of each strain were taken before and after the treatment and the GTP-Rho1p was pulled down from the cell extracts with GST-C21RBD and blotted against 12CA5, an anti-HA mAb. Total HA-Rho1p and HA-Rho4p was visualized with Western blotting.

Because *mtl2*Δ mutants have severe cell integrity defects which have been linked to defects in the Rho1p signaling pathway, we first examined whether upregulation of Rho1p or any of its activators (Rho1-GEFs) could suppress the growth defect of the *mtl2*Δ mutants in the presence of Csp. As shown in Figure 4A, moderate expression of *rho1*^+^, *rgf1*^+^, and *rgf2*^+^ restored the growth of an *mtl2*Δ mutant in the presence of the antifungal agent, whereas overexpression of *rgf3*^+^ did not suppress the growth defect. Similarly, we asked whether upregulation of the protein kinase C homologues Pck1p and Pck2p, both targets of Rho1p in *S. pombe*, restored the growth defect of an *mtl2*Δ mutant in the presence of Csp. Both gene products – *pck1*^+^ and *pck2*^+^ – share overlapping roles in cell viability (Toda et al. 1993); Pck2p also acts upstream from the Pmk1 MAPK signaling pathway (Ma et al. 2006). As shown in Figure 4A, only a moderate expression of *pck1*^+^ restored the growth of an *mtl2*Δ mutant in the presence of the antifungal agent. We also found that *wsc1*^+^ expressed from plasmids, containing the *wsc1*^+^ genomic promoter (pAL-*wsc1*^+^) (Fig. 4A) or the lower strength *P81xnmt1* promoter (pRZ17) (not shown), fully rescued the Csp hypersensitivity of *mtl2*Δ cells in rich medium.

To investigate whether *wsc1*Δ cells were defective in cell integrity signaling, we performed a suppression analysis of the *wsc1*Δ hypersensitive phenotype. Overexpression of Rho1p (pAL-*rho1*^+^) and Rgf1p (pAL-*rgf1*^+^) weakly suppressed the hypersensitivity of *wsc1*Δ cells to Csp (Fig. 4A). Moreover, the overexpression of Mtl2p did not suppress the *wsc1*Δ growth defect in the presence of the antifungal agent. In fact, *wsc1*Δ cells were sicker when Mtl2p was mildly overexpressed (OP) (not shown).

We also wondered whether the cell death produced by depletion of Mtl2p and Wsc1p could be overcome by activation of Rho1p or its regulators. As shown in Figure 4B, the activation of Rho1p, through the expression of a constitutively active form of Rho1p or overexpression of the wild-type Rho1p or Rgf1p, efficiently restored the growth of a strain (*P81nmt*-*mtl2 wsc1*Δ) unable to grow in the presence of thiamine (promoter off). The above findings strongly suggest that the essential cellular role of the putative sensors (Mtl2p and Wsc1p) is to activate Rho1p. Therefore, we analyzed the in vivo amount of GTP-bound Rho1p (activated-Rho1p) in cells with several different levels of Mtl2p and Wsc1p. In these experiments, GTP-bound Rho1p was obtained by precipitation with GST-C21RBD, the rhotekin-binding domain, in the extracts from the wild-type, the *mtl2*Δ and the *wsc1*Δ mutants, all of them containing HA-*rho1*^+^ expressed from its own promoter (see Experimental Procedures). Figure 4C shows that the amount of active Rho1p was similar in all three strains, suggesting that neither protein was necessary for the basal level of Rho1p-GTP under optimum growth conditions. Because hypersensitivity to Csp is often associated with defects in β–glucan synthesis, we tested whether Mtl2p or Wsc1p was required for the synthesis of a supplemental amount of Rho1p-GTP bound under stress conditions by measuring the level of Rho1p-GTP in cells that had been challenged with sublethal concentrations of Csp. Interestingly, *mtl2*Δ and *wsc1*Δ mutants contained much less Rho1p-GTP than wild-type cells when the cultures were grown in the presence of 0.1 μg/mL of Csp for 16 h prior to harvesting (Fig. 4C). Thus, it seems that both Mtl2p and Wsc1p are required to maintain the levels Rho1p under chronic cell wall stress.

### Functional relationship between Wcs1p and Rgf2p

Negative genetic interactions commonly occur between genes that function in parallel pathways to regulate the same essential function (Baryshnikova et al. 2010). The above results suggest that Mtl2p and Wsc1p are functionally related to preserve cell integrity in response to cell wall damage. The Mtl2p cell wall defects were readily suppressed by Wsc1p, Rgf1p, Rgf2p, Rho1p, and Pck1p, suggesting the existence of a defined pathway acting downstream from Mtl2p. However, the role of Wsc1p in this putative pathway or another pathway is to a large extent unknown.

**Figure 5:**
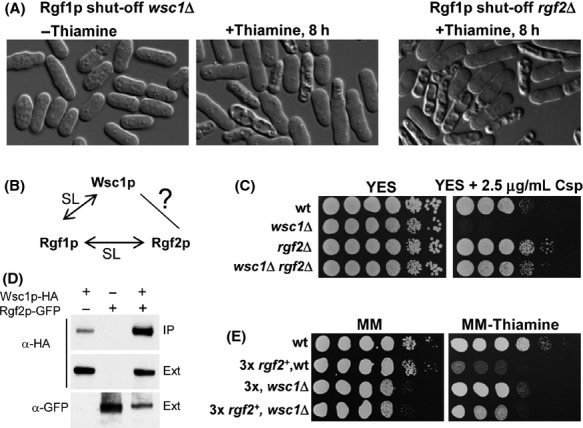
Interaction of Wsc1p and Rgf2p. (A) Depletion of Rgf1p in a *wsc1*Δ background leads to a lysis phenotype similar to the depletion of Rho1p. The *P81nmt-rgf1wsc1*Δ (SC173) and *P81nmt-rgf1rgf2*Δ (GRG33) shut-off mutants grown at 28°C in MM were supplemented with thiamine to repress the *nmt* promoter. Nomarski micrographs were taken before and after 8 h in MM with thiamine. (B) Diagram of the synthetic lethal interactions among *rgf1*^+^/*wsc1*^+^ and *rgf1*^+^/*rgf2*^+^. (C) Serial dilutions of wild-type (YS64), *wsc1*Δ (GRG15), *rgf2*Δ (PG1), and *wsc1*Δ *rgf2*Δ (SC98) cells grown on YES and YES plus 2.5 μg/mL of Csp at 28°C for 3 days. (D) Coprecipitation assay of Wsc1p and Rgf2p. Extracts from cells expressing Rgf2p and Wsc1p-HA; Rgf2p-GFP and Wsc1p; and Rgf2p-GFP and Wsc1p-HA were precipitated with anti-GFP and protein A-Sepharose beads and blotted against 12CA5 monoclonal anti-HA antibody. Whole-cell extract fractions (Ext) were assayed with anti-HA and anti-GFP antibodies. (E) Serial dilutions of wild-type and *wsc1*Δ cells transformed with pREP3X (empty plasmid) and pREP3X-*rgf2*^+^ (Rgf2-OP) grown on MM plates with thiamine (*nmt*, promoter off) or without thiamine (*nmt*, promoter on) at 28°C for 4 days. Overexpression of *rgf2*^+^ causes cell growth arrest in wild-type cells but not in *wsc1*Δ cells.

To examine whether Wsc1p acted on Rho1p activation through any of its GEFs, we analyzed the localization of Rho-GEFs in *wsc1*Δ and wild-type cells. Rgf1p, Rgf2p, and Rgf3p stimulate nucleotide exchange activity toward Rho1p and localize to different areas of the cortex; Rgf1p localizes to the cell tips in interphase cells and to the division septum in mitotic cells. Rgf2p is specific to the forespore inner membrane and Rgf3p localizes exclusively to the septum region of the cell (Tajadura et al. 2004; Morrell-Falvey et al. 2005; Mutoh et al. 2005; García et al. 2006a, 2009b). There was no appreciable difference in the localization of Rgf1-GFP, Rgf2p-GFP, or Rgf3p-GFP in *wsc1*Δ cells as compared to wild-type cells (Fig. S3), suggesting that Wsc1p is not required for the proper localization of any of the Rho1p-GEFs. Suppression of Csp hypersensitivity in *mtl2*Δ cells is carried out by Rgf1p and Rgf2p but not Rgf3p and hence we assumed that Wsc1p could be more related to these proteins than to Rgf3p. We deleted Wsc1p in *rgf1*Δ and *rgf2*Δ cells to conduct epistasis experiments. The *wsc1*Δ*rgf2*Δ double mutant was viable, but we failed to find any double-mutant spore *wsc1*Δ*rgf1*Δ. This result was confirmed by looking at the phenotype of the shut-off strain, *P81nmt*-*rgf1 wsc1*Δ, deleted for *wsc1*^+^ and with the endogenous *rgf1*^+^ promoter replaced by the *P81nmt* promoter. The strain was viable in the absence of thiamine (promoter on) but it could not form colonies in the presence of thiamine (promoter of) (not shown). In the shut-off experiments, a high percentage of the cells lysed as long cells (Fig. 5A), a phenotype characteristic of *rgf1*Δ mutants also seen in *P81nmt*-*rgf1 rgf2*Δ (Fig. 5A) and in *P81nmt*-*mtl2 wsc1*Δ (Fig. 2A). The Rgf1p/Rgf2p pair (García et al. 2009b) and the Rgf1p/Wsc1p pair of proteins share an essential function during vegetative growth and we therefore surmised that the Wsc1p and Rgf2p pair might also be functionally related (Fig. 5B). To test this hypothesis, we looked at the phenotype of the single and the double mutant. The *rgf2*Δ, *wsc1*Δ, and *wsc1*Δ*rgf2*Δ cells grew well at different temperatures (25, 28, 32, and 37°C). However, regarding the Csp hypersensitivity phenotype, *rgf2*Δ was epistatic to *wsc1*Δ as the double-mutant *wsc1*Δ*rgf2*Δ grew like the single *rgf2*Δ mutant in the presence of the drug (Fig. 5C). This suggests that Wsc1p acts upstream of Rgf2p and it might play a direct role in regulating Rgf2p activity, perhaps by binding to Rgf2p. Accordingly, we performed coprecipitation experiments using extracts from cells carrying an HA epitope-tagged Wsc1p and Rgf2p-GFP (García et al. 2009b) expressed from their own promoters in plasmids. The extracts were immunoprecipitated with anti-GFP and protein A-Sepharose. As shown in Figure 5D, a band corresponding to Wsc1p-HA was enriched in the immunoprecipitate. These results indicate that Wsc1p binds to Rgf2p. However, it is still uncertain whether it acts as an activator or an inhibitor of Rgf2p signaling. In this sense, we found that while the overexpression of Rgf2p in the wild-type is lethal (García et al. 2006a), its overexpression in a *wsc1*Δ background was viable (Fig. 5E), suggesting that full activation of Rgf2p requires Wsc1p.

### High levels of Wsc1p elicit an aberrant morphology and increase β-(1,3)-glucan synthesis

**Figure 6:**
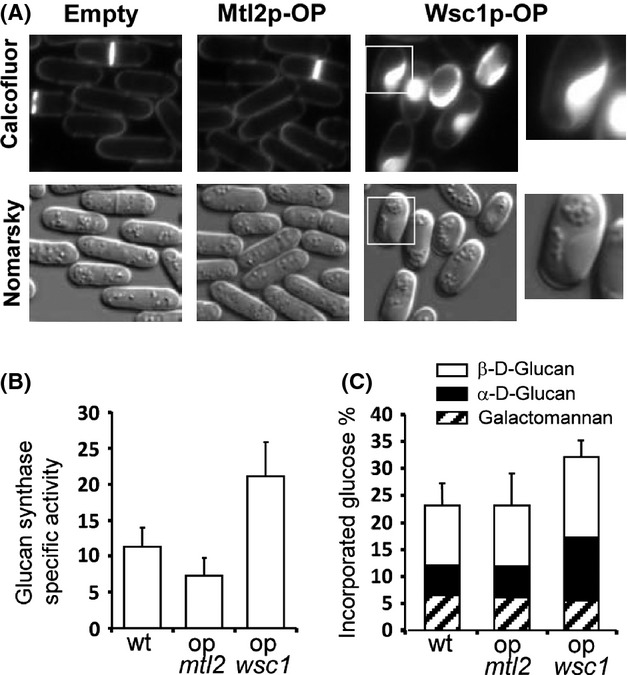
Overproduction of *wsc1*^+^ (OP) results in abnormal accumulation of cell wall material. (A) Nomarski and Cfw-stained UV micrographs of wild-type cells (Empty), w*sc1*^+^-overexpressing cells (Wsc1p-OP), and *mtl2*^+^-overexpressing cells (Mtl2p-OP) grown without thiamine for 20 h. The overexpression of *wsc1*^+^ causes cell growth arrest and morphological abnormalities. The Cfw fluorescence was concentrated in big patches. (B) In vitro GS activity assayed with the membrane fraction of the same strains described in A. All extracts were prepared from cells grown at 28°C in minimal medium without thiamine for 24 h. Values are means of at least three independent experiments with duplicate samples, and error bars represent standard deviations (SDs). (C) The level of glucan in cells overexpressing *mtl2*^+^ and *wsc1*^+^. Cells from the strains described in A were grown in the absence of thiamine for 24 h and [^14^C]-glucose was added 10 h before the cells were harvested. The graph represents the relative levels of [^14^C]-glucose radioactivity incorporated into each cell wall polysaccharide. Values are means of three independent experiments with duplicate samples. SDs for total carbohydrate values are shown.

In light of the above we reasoned that if Wsc1p was acting through Rgf2p and Rgf2p is involved in β-glucan biosynthesis it would be expected that Wsc1p overexpression would produce similar phenotypes to those of Rgf2p- or Rho1p-overexpressing cells. Constitutively active *rho1* mutants (Arellano et al. 1997) or the overexpression of the Rgf1p and Rgf2p genes cause an aberrant morphology and depositions of Cfw-stainable material (García et al. 2006a, 2009b). Thus, we analyzed the growth and the morphology of wild-type cells transformed with pREP3X-*wsc1*^*+*^ and pREP3X (empty) in the presence or absence of thiamine. In addition, we OP *mtl2*^+^ under the same conditions (pREP3X-*mtl2*^+^). When thiamine was eliminated to enhance *wsc1*^+^ or *mlt2*^+^ expression, the cells were unable to grow on plates (not shown). After 20 h of induction, Mtl2p overexpression produced long cells, probably due to cell separation defects, but otherwise their morphology was like that of the wild-type cells (Fig. 6A). However, cells overexpressing Wsc1p were round or misshapen and showed a general increase in Cfw fluorescence, most of them containing aberrant depositions (see enlarged cells in Fig. 6A, right). As expected, GS activity increased during *wsc1*^+^ overexpression. This activity was twofold higher than that observed in the wild-type strain (Fig. 6B). To corroborate these results, we also analyzed the cell wall composition of cells that OP *wsc1*^+^ or *mtl2*^+^. An increase was seen in the amount of β-glucan in cells overexpressing *wsc1*^+^ as compared with wild-type and Mtl2p-OP cells (15%, 11%, and 11.5%, respectively) (Fig. 6C) and this increase was similar to that seen in cells that OP *rho1*^+^ (15%) (Tajadura et al. 2004). There was also a general increase in cell wall biosynthesis in cells overexpressing *wsc1*^+^ as compared with wild-type cells or *mtl2*^+^ cells (32%, 23.5%, and 23%, respectively). In these cells, the α-glucan fraction was twofold that found in the wild-type *S. pombe* cells, indicating a simultaneous increase in both α- and β-glucan polymers (Fig. 6C). Additionally, the amount of galactomannan was not significantly affected. These results clearly indicate that Wsc1p is involved in the regulation of β(1,3)-glucan biosynthesis.

### Mtl2p and Wsc1p are not essential for Pmk1p signaling after cell wall stress

**Figure 7:**
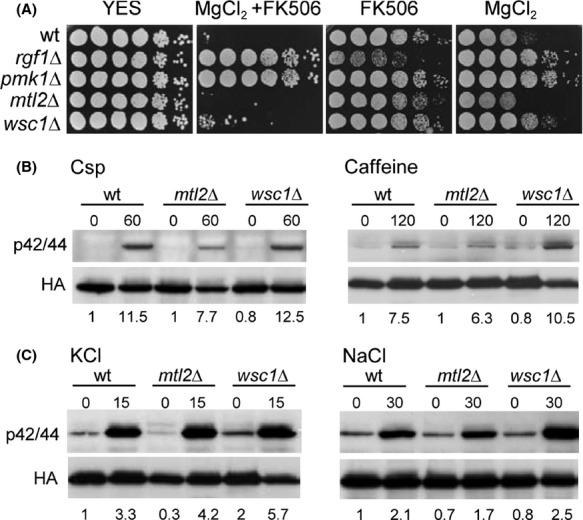
(A) *mtl2*Δ and *wsc1*Δ cells do not show the *vic* phenotype. Wild-type (YS64), *rgf1*Δ (VT14), *pmk1*Δ (MS192), *mtl2*Δ (YS1220), and *wsc1*Δ (SC136) were dropped onto the plates as indicated and then incubated for 4 days at 28°C. (B) Strains MI200 (*pmk1-HA6H*, control), SC19 (*pmk1-HA6H*, *mtl2*Δ), and SC141 (*pmk1-HA6H*, *wsc1*Δ) were grown in YES medium to mid log phase and treated with 1 μg/mL of Csp and grown for 60 min or treated with 15 mmol/L caffeine and grown for 2 h. Pmk1-HA6H was purified by affinity chromatography under native conditions from samples with or without treatment. Activated and total Pmk1p were detected by immunoblotting with anti-phospho-p42/44 or anti-HA antibodies, respectively. (C) The same experiments described in A, except that the cells were subjected to 0.6 mol/L KCl for 15 min or 0.5 mol/L NaCl for 30 min. Each treatment was repeated at least three times. Relative units comparing the induction-fold of wild type and the mutant cells in each individual experiment are shown below.

In *S. cerevisiae*, activated sensors initiate the signaling cascade that eventually activates a MAPK (Slt2p) by local recruitment of Rom2p to sites near the cell surface where cell wall remodeling is required (Lessage and Bussey 2006; Levin 2011). Activation of Slt2p triggers the phosphorylation of the transcriptional regulators Rlm1p and Swi4p/Swi6p, which regulate the transcription of cell wall synthesis-related genes. Fission yeast Rgf1p is the closest relative to Rom2p and regulates MAPK Pmk1p phosphorylation in response to the osmotic stress and cell wall stress triggered by Csp (Garcia et al. 2009a). We therefore examined the possibility that Mtl2p or Wsc1p could contribute to the regulation of the Pmk1p MAPK integrity pathway [cell wall integrity (CWI)]. First, we explored Pmk1p signaling by analyzing whether the *mtl2*Δ or *wsc1*Δ mutants showed the *vic* phenotype (viable in the presence of immunosuppressant and chlorine ions) characteristic of knockouts of components of the CWI pathway (Ma et al. 2006). Inhibition of calcineurin activity by FK506 results in MgCl_2_ hypersensitivity in fission yeast, and the elimination of the cell integrity MAPK components, such as Mkh1p, Rho2p, or Rgf1p, suppresses hypersensitivity to Cl^−^, allowing the cells to grow under these conditions (Sugiura et al. 1998, 1999). Like the wild-type cells, *mtl2*Δ cells did not show the *vic* phenotype and were unable to grow in the presence of MgCl_2_ and FK506, while *wsc1*Δ showed a very mild *vic* phenotype (Fig. 7A). Although these experiments suggested that neither Mtl2p nor Wsc1p was directly involved in the regulation of the Pmk1p pathway, we further explored this possibility by measuring with a p44/42 antibody the basal- and stress- induced level of Pmk1p phosphorylation in control, *mtl2*Δ and *wsc1*Δ cells that express a chromosomal HA6H-tagged version of Pmk1 (Madrid et al. 2006; Barba et al. 2008). The *mtl*2Δ cells showed hypersensitivity to Csp, caffeine, and sodium vanadate (Figs. 1, S1), whose effects have been related to changes in the architecture of the yeast cell wall. Therefore, we first analyzed Pmk1p activation in the wild-type and the mutant cells subjected to cell wall stress. As shown in Figure 7B, *mtl2*Δ and *wcs1*Δ cells displayed a marked increase in Pmk1p activity after Csp treatment, very similar to that seen in control cells. In addition, Pmk1p phosphorylation was not affected very much by deletion of the *mtl2*^+^ or *wsc1*^+^ after caffeine (15 mmol/L, 2 h) (Fig. 7B), sodium vanadate (5 mmol/L, 2 h), and oxidative stress (6 mmol/L hydrogen peroxide, 15 min) treatments (not shown). The level of Pmk1 phosphorylation increases quickly and transiently when *S. pombe* cells are subjected to a salt-induced osmostress caused by KCl and NaCl. This treatment may serve to reinforce the cell membrane and cell wall structure to withstand the physical challenge. However, as shown in Figure 7B, the presence of Mtl2p and Wsc1p was dispensable for Pmk1p activation during treatment with KCl and NaCl, suggesting that neither Mltl2p nor Wsc1p play a significant role in the signaling cascade that modulates MAPK activation under osmotic stress.

Finally, we wondered whether the hypersensitivity to Csp of the *mtl2*Δ cells could be overcome by upregulation or activation of the cell integrity pathway driven by Pmk1p. We found that the MAPK Pmk1p/Spm1p under the control of the strong and constitutively active alcohol dehydrogenase promoter (Zaitsevskaya-Carter and Cooper 1997) was unable to suppress the hypersensitive phenotype of Csp in *mtl2*Δ mutants (Fig. 4A). Moreover, the introduction in the *mtl2*Δ strain of a constitutively active mutant *pek1*^*DD*^, which increases the tyrosine-phosphorylation of Pmk1p–HA (Sugiura et al. 1999), was unable to suppress the hypersensitivity to Csp in that strain (not shown). Taken together, our results support the view that Pmk1p signaling is not compromise in the *mtl2*Δ and *wcs1*Δ strains and suggest that the functional relevance of *S. pombe* sensor-like proteins in the CWI pathway differs from that of *S.cerevisiae*.

## Discussion

In fungi, the cell wall is the key defense to withstand environmental adversity. Due to damage inflicted by stressors and antifungal drugs, the cell wall is repaired and even fortified through cell wall biosynthesis. In recent years, the synthesis and the overall composition of the cell wall has been studied in fission yeast. However, little is yet known about the initial steps in cell wall stress sensing and how the sensors and regulatory components of the main polysaccharides cooperate to assemble the cell wall when exposed to suboptimal or hostile environments.

Here, we investigated two proteins with the characteristics of cell wall stress sensors and found that together they play an essential role in cell integrity. Both proteins act by turning on the Rho1p GTPase. However, the specific role of each of them is quite different; thus, while Mtl2p is involved in the detection of and reaction to different environmental changes (Fig. 2), Wsc1p is redundant regarding the response to stress but its overstimulation reinforces cell wall biosynthesis (Fig. 6). Cell growth in the presence of cell wall stress is critical for survival and hence our work sheds further light on the involvement of Rho1p signaling in the control of morphogenesis under stressful conditions.

Fission yeast *mtl2*^+^ is crucial for maintaining cell wall integrity under different cell wall stresses. We report Mtl2p as a novel upstream component of the Rho1p/Pck1p signaling pathway. First, deletion of the *mtl2*^+^ gene in vegetative cells caused a modest degree of cell lysis, with a very similar morphology to those of cells devoid of Rho1p or Pck1p/2p activity. Consistent with this, *mtl2*Δ cells were defective in GS activity and showed a lower amount of β–glucan, suggesting that one of the main functions of Mtl2p would be the regulation of GS activity. Second, *mtl2*Δ cells were hypersensitive to cell wall antifungal drugs and this hypersensitivity was suppressed by mild overexpression of *rho1* (Fig. 4) but not of any of the other *rho* genes (not shown). Expression of the Rho1p GEFs *rgf1*^+^ and *rgf2*^+^ and an effector of Rho1p, *pck1*^+^ rescued the growth defect in the presence of Csp, further supporting the notion that Mtl2p functions upstream Rho1p (Fig. 4). Third, biochemical data strongly support the view that Mtl2p acts as an upstream regulator of Rho1p. Extracts from *mtl2*Δ cells were deficient in catalyzing the GTP-loading of Rho1p in the presence of chronic stress caused by a sublethal concentration of Csp, while they showed wild-type levels of GTP-Rho1p in the absence of cell wall stress. Accordingly, Mtl2p is required for the viability of yeast cells during vegetative growth under stress conditions that compromise cell wall integrity, including caffeine, orthovanadate, and oxidative stress.

Disruption of Wsc1p caused no obvious defect in cell wall composition except for a minor cell lysis defect which was exacerbated when combined with shut-off of the *mtl2*^+^ gene (Fig. 2). This could be due to a functional overlap of the two sensors in the maintenance of cellular integrity. Moreover, it is possible that Mtl2p could stimulate basal Rgf1p/Rgf2p/Rho1p/Pck1p signaling, whereas Wsc1p could be involved in the maintenance of cellular integrity during extreme situations, such as a higher amount of Csp (>3 μg/mL). Indeed, when *mtl2*Δ cells were plated on 2 μg/mL of Csp the mutant grew extremely slowly. Nonetheless, a significant number of the *mtl2*-null cells gave rise to small colonies, while no *mtl2*-null cells survived at 0.5 μg/mL of Csp (Fig. 1C). These results suggest two important conclusions: (1) Mtl2p is necessary for growth at low doses of Csp but is not essential for growth at high doses of Csp and (2) there are fundamental differences in the cellular response to low levels of Csp and high levels of Csp in fission yeast, which has been documented as the paradoxical effect in diverse yeast species (Wiederhold et al. 2005). In addition, each sensor could be regulating different modes of stress response, for example, restraining the action of cell wall degrading enzymes or reinforcing the cell wall by the enhanced production of α-glucans and β-glucans (Martin et al. 2003; Cortes et al. 2005). While disruption of each individual sensor could be overcome by reinforcement of the cell wall with components dependent of the other, disruption of both sensors might produce blockage on both salvage pathways compromising cell integrity and causing cell lysis (Jendretzki et al. 2011).

Here, we found that overexpression of Wsc1p reinforced the cell wall (Fig. 6A) and activated β-1,3 glucan biosynthesis, probably through activation of β-GS by Rho1p. Furthermore, we present evidence that the Wsc1p activation of Rho1p is mediated by Rgf2p, although we cannot exclude a role for Rgf1p in the process. We hypothesize that in order to support high doses of cell wall stress the wild-type cells activate Rgf2p, which in turn competes with Rgf1p and blocks Rgf1p binding to Rho1p. However, in the absence of Wsc1p, Rgf2p is not active and hence the signal is not transmitted and the cells die. In a similar situation, but in the absence of Rgf2p, Rgf1p – which is activated by other means, probably Mtl2p – could freely bind to Rho1p and the cells would remain alive in the presence of high concentrations of Csp. In the absence of both sensors, *wsc1*^+^ and *mtl2*^+^, the signals are not transmitted to Rgf1p and Rgf2p and the cells die. Contrary to Wsc1p, the overexpression of Mtl2p had little effect on β-1,3 glucan synthesis. Our results are consistent with cell wall assembly being regulated by two distinct networks involving Rho1p, at least in stress situations. One involves signaling from Mtl2p through Rho1p to Pck1p, and the second involves specific signaling from Wsc1p and Rgf2p through Rho1p to activate GS and cell wall biosynthesis.

In fission yeast, the so-called “cell integrity” pathway contains a module of three kinases – Mkh1p, Skh1p/Pek1p, and Pmk1p/Spm1p-, and the absence of any of these components induces different phenotypes involving defects in cytokinesis and vacuole fusion, hypersensitivity to potassium ions, and increased lysis after β-glucanase treatment (Toda et al. 1996b; Zaitsevskaya-Carter and Cooper 1997; Sugiura et al. 1999; Loewith et al. 2000). Rho2p and Rgf1p act upstream Pck2p, regulating the Pmk1p MAPK pathway in response to osmotic and cell wall stress (Ma et al. 2006; Garcia et al. 2009a). Pck1p negatively regulates Pmk1p basal activity (Barba et al. 2008) while Rgf2p involvement in CWI signaling has been analyzed, but with negative results (García et al. 2009b).

We found that neither a deletion of Mtl2p nor deletion in Wsc1p generates defects in the CWI pathway. First, knockout of Mtl2p and Wsc1p did not elicit the *vic* phenotype characteristic of the mutations in components of the CWI pathway. Second, the overexpression of *rho2*^+^ (not shown), *pck2*^+^, or the hyperactivation of Pmk1 MAPK by the expression of the constitutively active *Pek1*^DD^ did not suppress sensitivity to Csp in the *mtl2*Δ cells. Third, Pmk1p signaling remained active in *mtl2*Δ and *wsc1*Δ disruptants exposed to cell wall stress and osmotic stress (Fig. 7). Fourth, we examined the level of Pmk1p phosphorylation upon cell wall stress in *P81nmt*-*mtl2 wsc1*Δ cells repressed with thiamine for 4 h. We found that even when more than 30% of the cells had shrunk (Fig. 2A), cells still responded to the Csp treatment by activating Pmk1p (not shown). Finally, in a global map of genetic interactions (Ryan et al. 2012) have shown that Mtl2p clustered with Pmp1p (the phosphatase that downregulates Pmk1p), Pck1p (a negative regulator of CWI pathway), Yam8p and Cch1p (both encoding putative subunits of a Ca^2+^channel required for the stimulation of calcineurin) and Rga8p (a Rho1p GAP). None of these genes is involved in Pmk1p activation.

Our results therefore suggest that the cell wall stress-sensing spectrum of *S. pombe* sensor-like proteins differs from that of *S.cerevisiae*. This line of reasoning is supported by a report indicating that the central components of the osmotic, oxidative, and cell wall stress signaling pathways are relatively well conserved, whereas the sensors and transcriptional regulators of these modules have diverged significantly (Nikolaou et al. 2009). Further investigation of the specificity of signals upstream from Rho1p effectors should provide a key to answering how the orchestration of small GTPase protein functions can be achieved.

## Supporting Information

**Figure S1.** The *mtl2*Δ cells are hypersensitive to different types of cell wall stress. Equal numbers of wild-type, *wsc1*Δ and *mtl2*Δ cells were diluted and (4 × 10^4^, 2 × 10^4^, 2 × 10^2^ and 2 × 10^1^ cells respectively) were spotted onto YES plates without and with caffeine (15 mmol/L), sodium orthovanadate (1.7 mmol/L), SDS (0.015%), hydrogen peroxide (0.8 mmol/L) and NaCl (100 mmol/L). Colony formation was analysed after 3 days of incubation at 28ºC.

**Figure S2.** Localization of Wsc1p-GFP and Mtl2p-GFP to the cortex is not altered by microtubule depolymerisation. (A) Cells expressing Wsc1p-GFP and mCherry-atb2 (SC240) were treated with dimethyl sulfoxide (1%) (upper panels) or 25 μg/mL MBC (lower panels) for 20 min and then analysed for Wsc1p-GFP localization (left), mCherry-atb2 (middle) or Nomarski (right). (B) Cells expressing Mtl2p-GFP and mCherry-atb2 (SC242) were treated and analysed as above.

**Figure S3.** The distribution of Rgf1p-GFP, Rgf2p-GFP and Rgf3p-GFP is not affected in strains deleted for *wsc1*^+^. (Upper panel) localization of Rgf1p-GFP in wild-type (PG40) and *wsc1*Δ cells (SC165). (Middle panel) localization of Rgf2p-GFP, in wild-type (HVP54 + p41X-Rgf2-GFP) and *wsc1*Δ cells (SC136+ p41X-Rgf2-GFP). (Bottom panel) localization of Rgf3p-GFP in wild-type (SC238) and *wsc1*Δ cells (SC198). Cells were grown to log phase in YES medium at 28ºC.
