# Supplementary figures and images for: The fission yeast cell wall stress sensor-like proteins Mtl2 and Wsc1 act by turning on the GTPase Rho1p but act independently of the cell wall integrity pathway
Source: Microbiologyopen. 2013 Jul 30;2(5):778–94. doi: 10.1002/mbo3.113 (PMC3831639; doi:10.1002/mbo3.113)

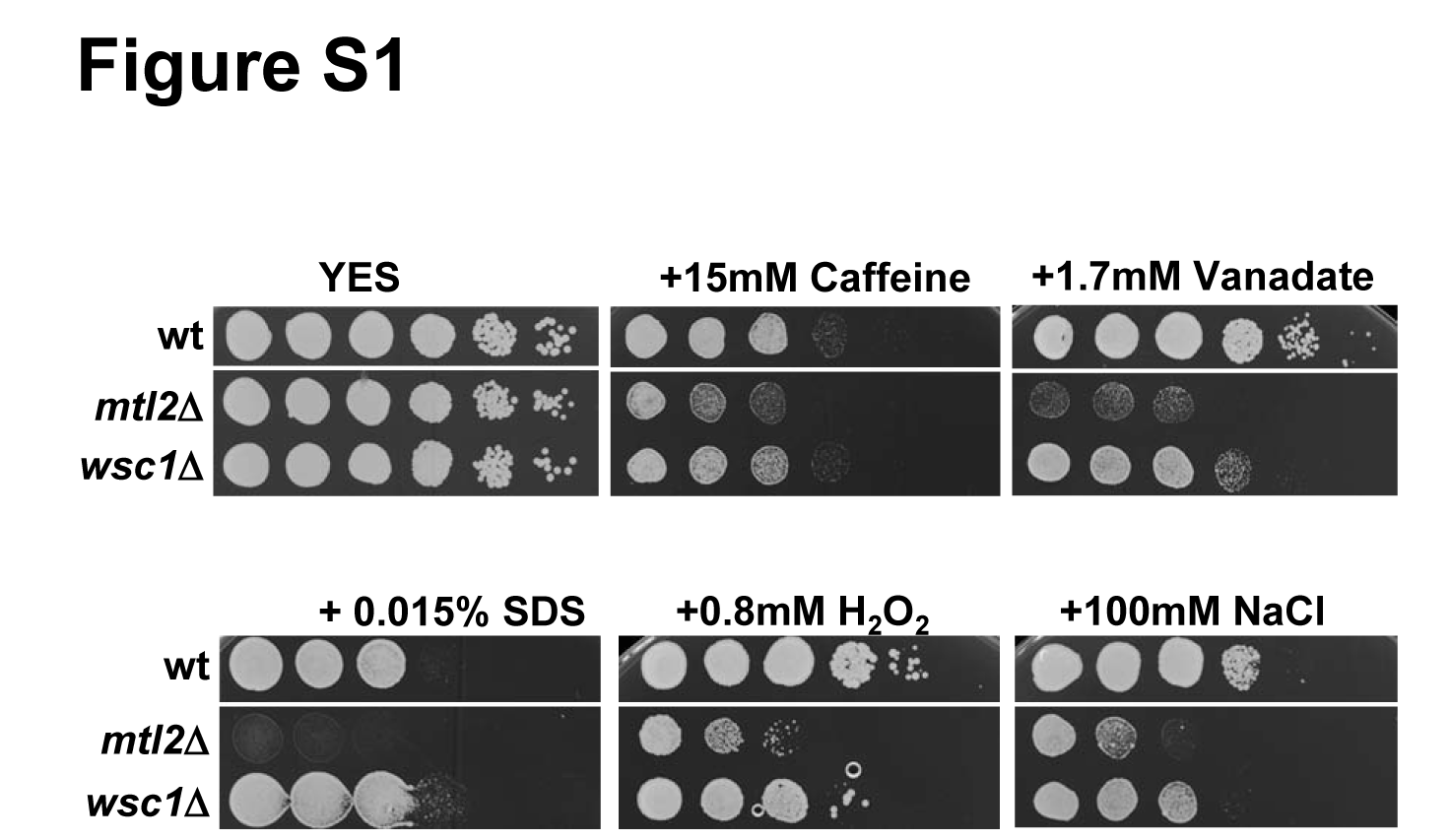

Supplement: Supplementary file 1 [file mbo30002-0778-SD1.tif]

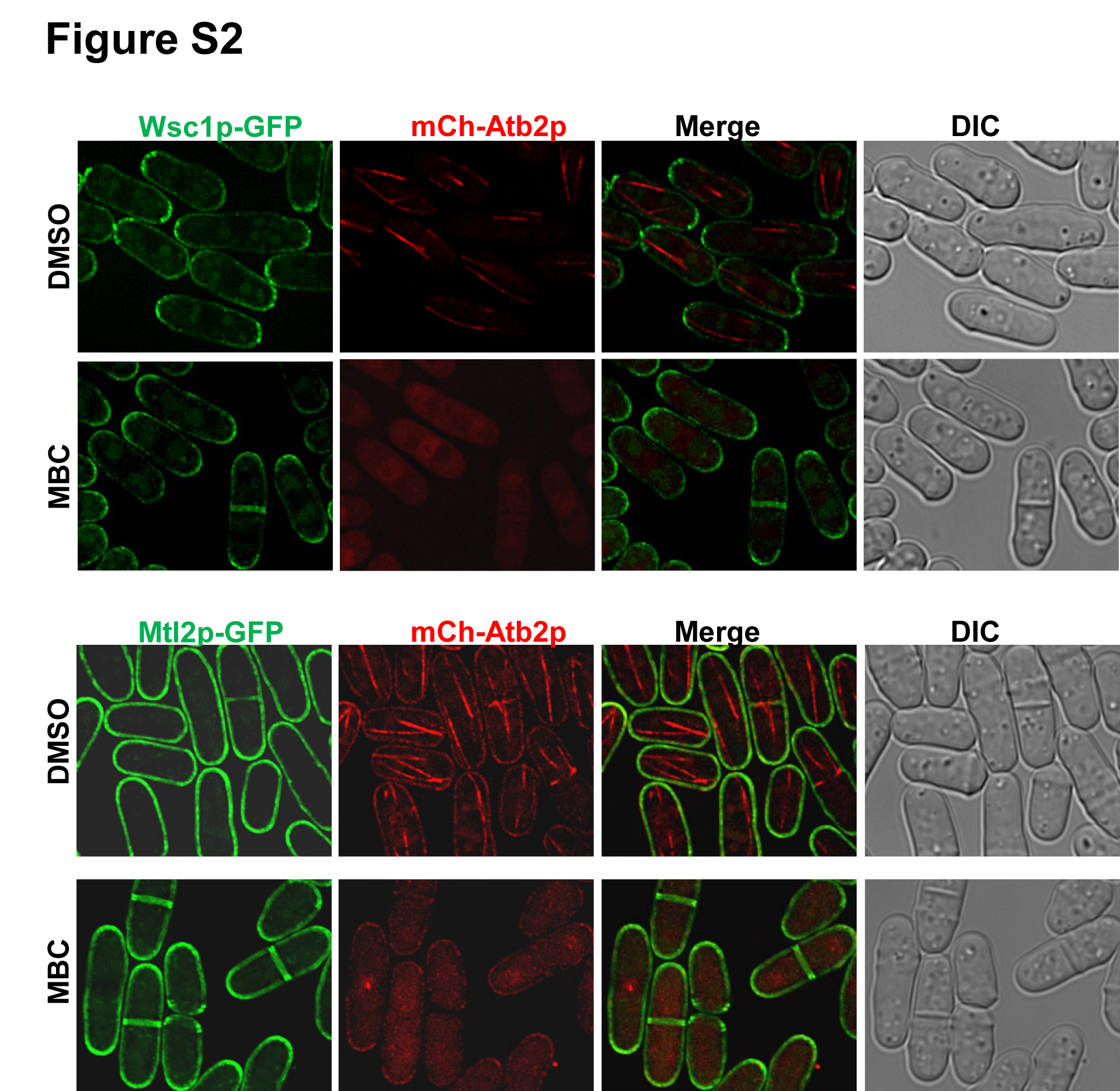

Supplement: Supplementary file 2 [file mbo30002-0778-SD2.tif]

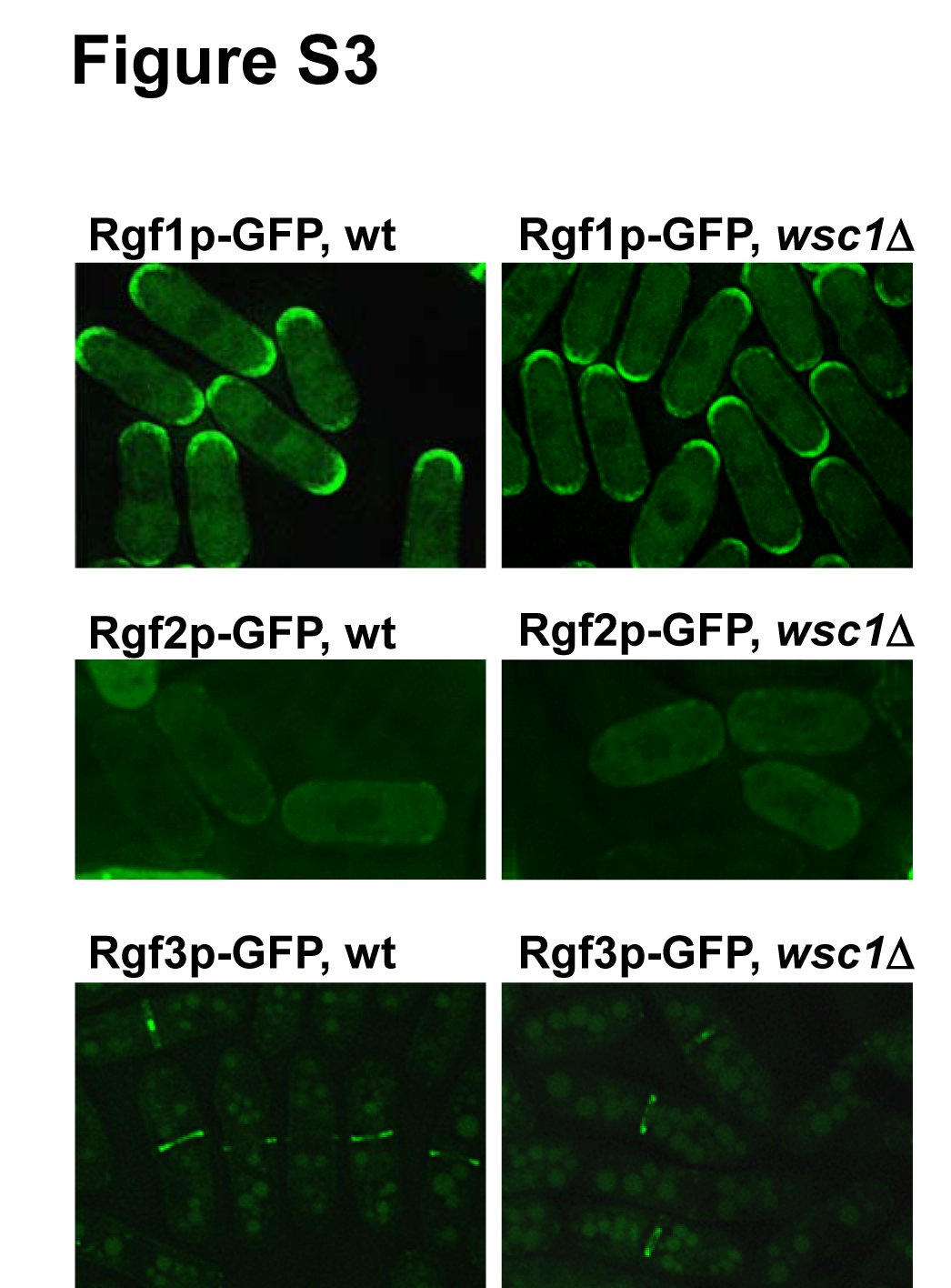

Supplement: Supplementary file 3 [file mbo30002-0778-SD3.tif]
